# Revolutionizing scar management: a compelling case report on the efficacy of manual fractional photothermolysis for treating mature burn scar contractures: Case report

**DOI:** 10.1097/MD.0000000000047500

**Published:** 2026-01-30

**Authors:** Jianfeng Yang, Pan Xiao, Bin Liu

**Affiliations:** a Department of Burn and Plastic Surgery, Xiangtan Central Hospital, Xiangtan, Hunan, China; b Graduate Collaborative Training Base of Xiangtan Central Hospital, Hengyang Medical School, University of South China, Hengyang, Hunan, China.

**Keywords:** contracture, fractional photothermolysis, function, scar

## Abstract

**Rationale:**

This case report introduces manual fractional photothermolysis (MFP) as a novel, noninvasive technique for managing burn scar contractures that are refractory to conventional physical therapy, highlighting its potential to improve functional outcomes.

**Patient concerns:**

A 52-year-old man presented with severe functional limitation of the 2nd, 3rd, and 4th digits of his left hand due to burn scar contractures, which showed no improvement after more than 6 months of intensive physical therapy.

**Diagnoses:**

The patient was diagnosed with postburn scar contractures of the left hand (7 months post-injury).

**Interventions:**

The patient underwent treatment with the novel MFP technique.

**Outcomes:**

Objective assessment by 2 independent physical therapists confirmed an improved active range of motion post-intervention. The patient reported significant functional gains and high satisfaction. No complications were observed.

**Lessons:**

This case suggests that MFP is a safe and effective intervention for improving hand function in patients with therapy-resistant scar contractures. The technique, leveraging principles of fractional photothermolysis, holds promise for transforming clinical management strategies for similar conditions.

## 1. Introduction

Approximately 11 million people worldwide require medical care for burn injuries each year.^[1]^ Scar contractures across joints are a common complication of burns. Conventional management typically includes pressure garments, massage, physical therapy, and steroid injections, while reconstructive surgery is often needed for severe cases. The introduction of fractional laser devices in 2004^[2]^ marked a major advancement in scar treatment through fractional photothermolysis (FP). Professor Jun Tan’s seminal work clarified the mechanism of FP, showing that fractional lasers create microthermal zones (MTZs) that induce controlled microscopic thermal injury while preserving surrounding tissue, thereby promoting uniform epidermal and dermal remodeling. This technique has become widely recognized for treating surgical and burn scars.

However, conventional FP has limitations in addressing hypertrophic scars because its MTZs are relatively narrow and do not penetrate deeply enough. To overcome these shortcomings, Professor Tan developed manual fractional photothermolysis (MFP), a technique that uses an ultrapulse carbon dioxide laser to create intermittent microperforations in scar tissue. This approach improves scar pliability and has produced promising clinical results in hypertrophic scar management.^[3]^ Building on these findings, the present report evaluates the use of MFP to improve hand function in a patient with postburn scar contractures.

## 2. Materials and methods

### 2.1. Case presentation

A 52-year-old Chinese man sustained burn injuries to his left hand in a car accident, resulting in scars that matured over 7 months. The volar surfaces healed by secondary intention, while the small (5th) finger required amputation at the phalanx level. Despite over 6 months of intensive physical therapy, the patient developed persistent contractures of the index, middle, and ring digits. When further rehabilitation produced no additional functional gains, he was referred for surgical evaluation. Instead of surgery, we offered MFP as a less invasive alternative, and the patient agreed to proceed.

This treatment was approved by the Xiangtan Central Hospital Internal Review Board (IRB Approval No.: 2022-07-006). Written informed consent was obtained.

### 2.2. Methods

Tumescent anesthesia was administered before treatment. Using the ultrapulse mode (20 W, pulse width 0.3 ms, interval 1 ms) of a carbon dioxide laser (JLT-100B, Jin Lai Te Medical, Wuhan, China), we manually created MTZs across the contracted scar tissue. MTZs were applied selectively to the volar surfaces of the affected digits without circumferentially surrounding the proximal interphalangeal (PIP) joints. The diameter of each MTZ was maintained at 0.3 to 0.5 mm, with depth limited to the full thickness of the scar. Spacing between MTZs ranged from 1.5 to 3 mm. To minimize the risk of new scar formation, MTZ diameters did not exceed 0.5 mm.^[4]^ No cooling device was required.

Posttreatment, Moist Exposed Burn Ointment was applied every 4 to 6 hours until healing occurred. The procedure was repeated 1 month later. Functional rehabilitation exercises began immediately, and the patient adhered to a daily exercise program.

Treatment response was assessed by 2 experienced physical therapists, who measured the arc of motion (difference between maximal flexion and extension) of the PIP joints before and after treatment.

## 3. Results

**Figure 1.**
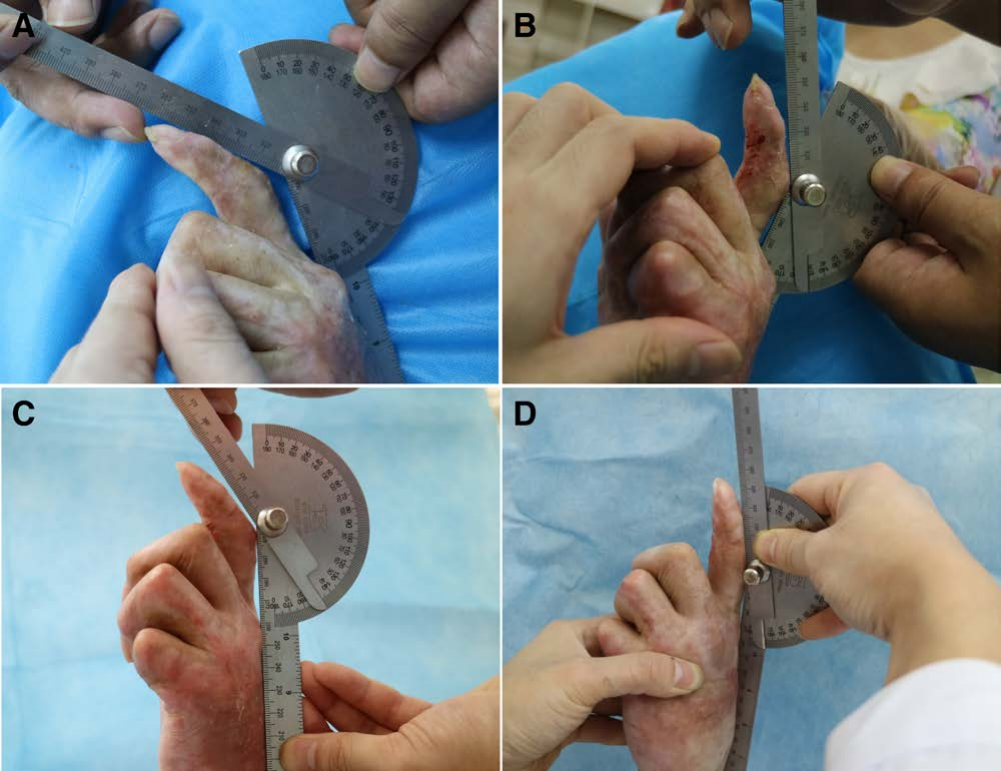
31° increase in active ROM of PIP joint for his 2nd digit (50°–81°). ROM = range of motion, PIP, proximal interphalangeal.

**Figure 2.**
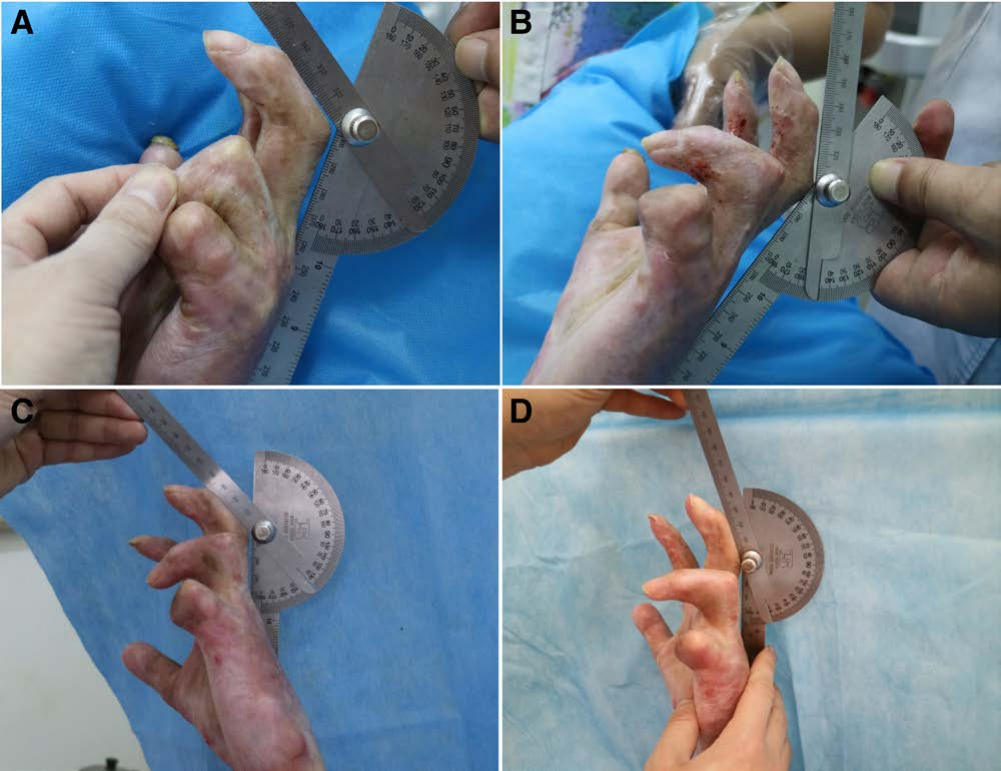
32° increase for his 3rd digit (38°–70°).

**Figure 3.**
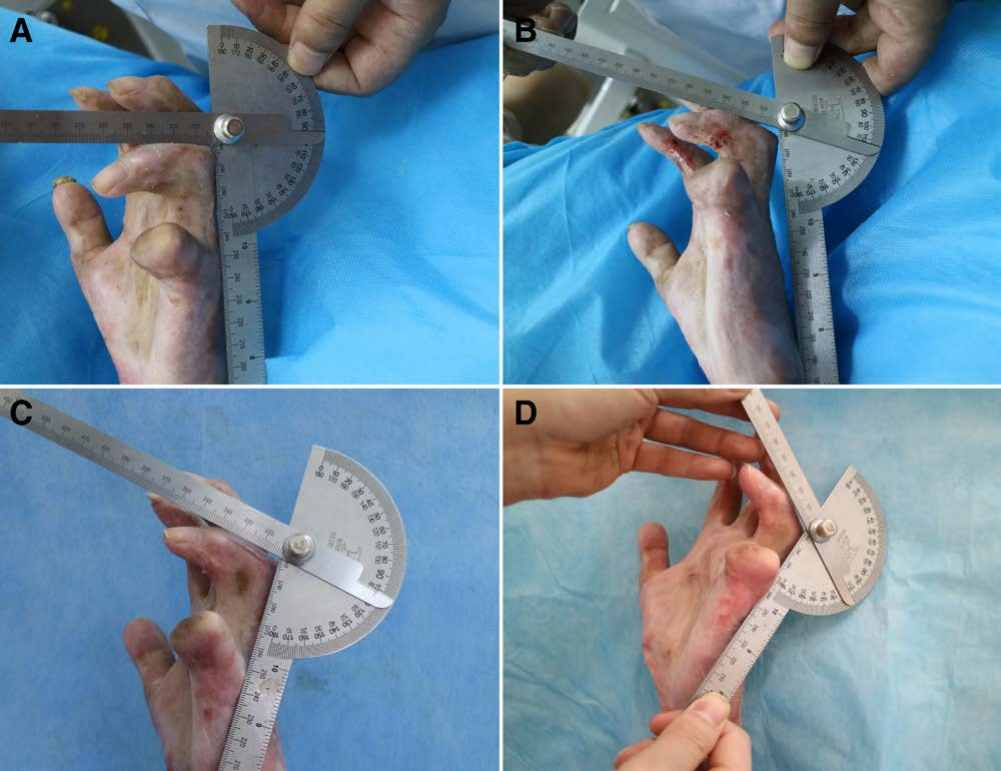
20° increase for his 4th digit (8°–28°).

Immediately after the procedure, the patient showed a significant increase in the active arc of motion of the PIP joints of the index, middle, and ring digits. A mild reduction in range of motion (ROM) occurred over the following days, despite ongoing physical therapy. Ultimately, ROM increased by 31° in the index finger (50°–81°) (Fig. 1), 32° in the middle finger (38°–70°) (Fig. 2), and 20° in the ring finger (8°–28°) (Fig. 3). Detailed ROM data are provided in Table 1.

**Table 1:** Pretreatment and posttreatment active ROM.

	Pretreatment	Immediately after	1 month after	Immediately after	3 months after
		1st treatment	1st treatment	2nd treatment	2nd treatment
2nd digit	50	62	60		81
3rd digit	38	50	45		70
4th digit	8	17	13	20	28

**Figure 4.**
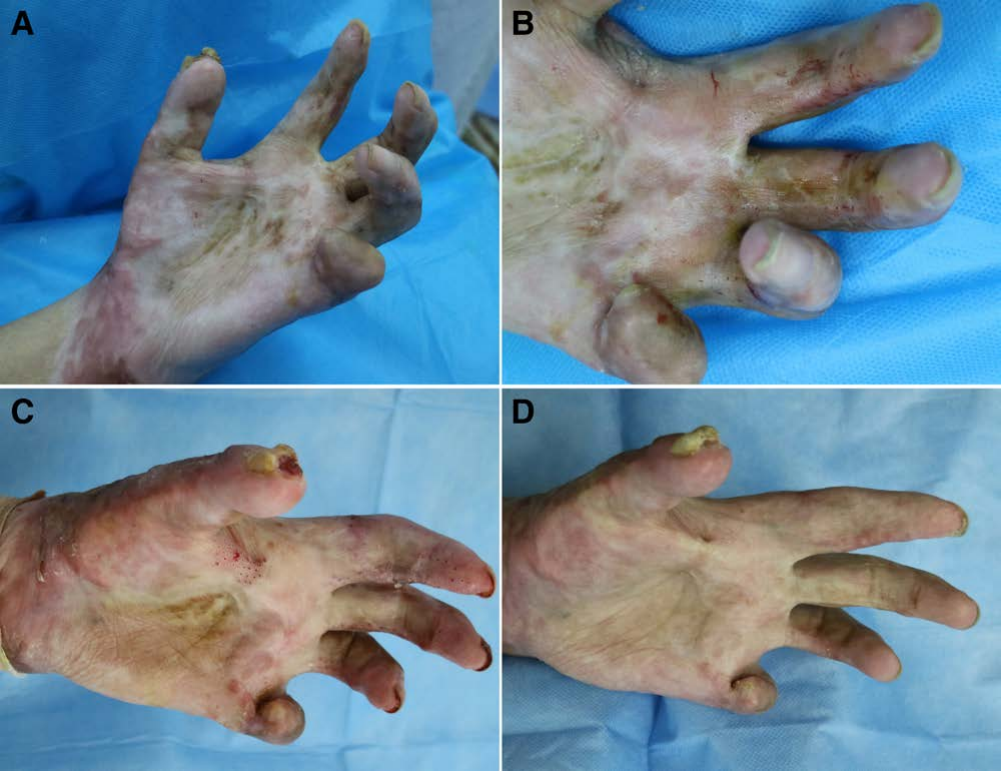
Three months after the second treatment, the patient was very satisfied with the function and appearance improvement of his left hand.

Three months after the second treatment, the patient expressed high satisfaction with both functional and aesthetic improvements (Fig. 4). He chose to discontinue further treatment due to financial concerns, believing that his hand function sufficiently met his daily needs. No complications, including infection, depigmentation, hyperpigmentation, or worsening scarring, were noted.

## 4. Discussion

Scar contractures across joints can severely restrict ROM and lead to significant functional impairment. Common treatments include massage, physical therapy, and reconstructive surgery.^[5,6]^ FP, introduced by Hantash et al^[7]^ in 2004, creates MTZs that promote rapid epithelial repair and dermal remodeling by preserving surrounding viable tissue. Numerous reports have demonstrated improvements in scar appearance, function, and symptoms after FP.^[8–11]^

In our experience, FP typically requires 3 or more sessions to achieve meaningful functional improvement, consistent with previous studies showing limited gains after a single treatment.^[12–17]^ To pursue more immediate and substantial functional recovery, we explored MFP in this case, which produced an immediate and notable increase in ROM, an effect not commonly reported with conventional FP.^[15–17]^ Experts attribute this early improvement to photomechanical scar release, similar to the enlargement of skin grafts after fenestration.^[16]^ We hypothesize that the larger diameter and deeper penetration of MTZs produced by MFP contribute to this enhanced effect.

Although a mild decline in ROM was observed in the following month, likely due to MTZ-related wound contraction, continued rehabilitation successfully preserved most of the functional gains. This underscores the importance of integrating functional therapy with MFP.

This patient ultimately achieved sufficient hand function to avoid reconstructive surgery. Without MFP, he might have required skin grafting to restore function. Thus, MFP may serve as a minimally invasive alternative for selected patients with scar contractures.

Nevertheless, careful evaluation is essential when applying this emerging technique. The optimal MTZ parameters suggested here are based on a single case and should be considered preliminary. While larger diameters, deeper penetration, and closer spacing may enhance scar release, we recommend limiting MTZ diameter to ≤0.5 mm, avoiding penetration beyond scar depth, and maintaining spacing >1.5 mm to reduce the risk of complications. Patient safety must remain the top priority. Further studies with larger cohorts are required to determine the safest and most effective treatment parameters for managing mature scar contractures.

## 5. Conclusion

To our knowledge, this is the first report of MFP as an effective and safe method for treating scar contractures. We believe that the combination of MFP and FP techniques may bring revolutionary changes to the management of scar contractures.

## Acknowledgments

The authors express their gratitude to Professor Jun Tan, Professor of the Department of Plastic Surgery and the Laser Aesthetic Center of the First Affiliated Hospital of Hunan Normal University, for his guidance in using MFP.

## Author contributions

**Conceptualization:** Jianfeng Yang, Bin Liu, Pan Xiao.

**Data curation:** Jianfeng Yang, Bin Liu, Pan Xiao.

**Formal analysis:** Jianfeng Yang, Bin Liu, Pan Xiao.

**Funding acquisition:** Jianfeng Yang, Bin Liu, Pan Xiao.

**Investigation:** Jianfeng Yang, Bin Liu, Pan Xiao.

**Methodology:** Jianfeng Yang, Bin Liu, Pan Xiao.

**Project administration:** Jianfeng Yang, Bin Liu, Pan Xiao.

**Resources:** Jianfeng Yang, Bin Liu, Pan Xiao.

**Software:** Jianfeng Yang, Bin Liu, Pan Xiao.

**Supervision:** Jianfeng Yang, Bin Liu, Pan Xiao.

**Validation:** Jianfeng Yang, Bin Liu, Pan Xiao.

**Visualization:** Jianfeng Yang, Bin Liu, Pan Xiao.

**Writing – original draft:** Jianfeng Yang, Bin Liu, Pan Xiao.

**Writing – review & editing:** Jianfeng Yang, Bin Liu, Pan Xiao.
